# Research on Crop Classification Using U-Net Integrated with Multimodal Remote Sensing Temporal Features

**DOI:** 10.3390/s25165005

**Published:** 2025-08-13

**Authors:** Zhihui Zhu, Yuling Chen, Chengzhuo Lu, Minglong Yang, Yonghua Xia, Dewu Huang, Jie Lv

**Affiliations:** 1 Department of Earth Science and Technology, City College, Kunming University of Science and Technology, Kunming 650093, China; zzh168@stu.kust.edu.cn (Z.Z.); 20130051@kust.edu.cn (M.Y.); 20040063@kust.edu.cn (Y.X.); dwhuang81@163.com (D.H.); 2 School of Geography and Information Engineering, China University of Geosciences (Wuhan), Wuhan 430078, China; ylchennn@cug.edu.cn; 3 School of Land and Resources Engineering, Kunming University of Science and Technology, Kunming 650093, China; 20232201100@stu.kust.edu.cn

**Keywords:** multimodal remote sensing, crop classification, Sentinel-1, Sentinel-2, temporal feature fusion, random forest, U-Net

## Abstract

Crop classification plays a vital role in acquiring the spatial distribution of agricultural crops, enhancing agricultural management efficiency, and ensuring food security. With the continuous advancement of remote sensing technologies, achieving efficient and accurate crop classification using remote sensing imagery has become a prominent research focus. Conventional approaches largely rely on empirical rules or single-feature selection (e.g., NDVI or VV) for temporal feature extraction, lacking systematic optimization of multimodal feature combinations from optical and radar data. To address this limitation, this study proposes a crop classification method based on feature-level fusion of multimodal remote sensing data, integrating the complementary advantages of optical and SAR imagery to overcome the temporal and spatial representation constraints of single-sensor observations. The study was conducted in Story County, Iowa, USA, focusing on the growth cycles of corn and soybean. Eight vegetation indices (including NDVI and NDRE) and five polarimetric features (VV and VH) were constructed and analyzed. Using a random forest algorithm to assess feature importance, NDVI+NDRE and VV+VH were identified as the optimal feature combinations. Subsequently, 16 scenes of optical imagery (Sentinel-2) and 30 scenes of radar imagery (Sentinel-1) were fused at the feature level to generate a multimodal temporal feature image with 46 channels. Using Cropland Data Layer (CDL) samples as reference data, a U-Net deep neural network was employed for refined crop classification and compared with single-modal results. Experimental results demonstrated that the fusion model outperforms single-modal approaches in classification accuracy, boundary delineation, and consistency, achieving training, validation, and test accuracies of 95.83%, 91.99%, and 90.81% respectively. Furthermore, consistent improvements were observed across evaluation metrics, including F1-score, precision, and recall.

## 1. Introduction

The continuous growth of the global population, coupled with climate change, has intensified the challenges to global food security. According to predictions by the Food and Agriculture Organization (FAO) of the United Nations, in order to meet the basic food needs of approximately 9.7 billion people by 2050, global food production must increase by about 60% compared to the levels in 2012 [1,2]. Against this backdrop, the timely and accurate acquisition of spatial distribution information of crops has become a crucial task for agricultural management and food security assessment. Traditional crop classification methods often rely on manual field surveys and statistical analyses, both of which are time-consuming and labor-intensive, and they lack detailed descriptions of the spatial distribution of crops. With its advantages of large coverage, high timeliness, and non-contact observation capabilities, remote sensing technology has become an important approach for achieving large-scale crop classification [3].

Optical remote sensing, such as Sentinel-2, is commonly used for the construction of vegetation indices (e.g., NDVI, NDRE) due to its rich spectral information, which can reflect the physiological status of crops. However, it is significantly affected by cloudy and rainy weather, making the acquisition of continuous time-series images challenging. Synthetic Aperture Radar (SAR) imagery, such as Sentinel-1, possesses all-weather imaging capabilities and can effectively complement the spatial and temporal gaps of optical images. It characterizes crop structural information through backscatter coefficients and polarization features (e.g., VV, VH). Nevertheless, radar images suffer from strong speckle noise, complex textures, and insensitivity to crop physiological characteristics, which affect their independent discriminative ability [4]. Despite existing studies demonstrating that deep learning methods, such as convolutional neural networks (CNN) and recurrent neural networks (RNN), have shown significant advantages in the feature extraction and classification of high-dimensional remote sensing images and that the fusion of optical and radar data can effectively enhance crop classification accuracy [5]. However, current methods still face the following three main challenges: first, feature selection strategies largely rely on empirical rules, making it difficult to adapt to complex surface changes; second, modality fusion approaches are mainly focused on data-level or decision-level fusion, with relatively insufficient research on feature-level fusion; third, although deep learning models demonstrate excellent performance, there remains a lack of systematic exploration of multimodal remote sensing feature construction and their collaborative optimization mechanisms.

To address the aforementioned shortcomings, this paper proposes a multimodal remote sensing crop classification method based on feature-level fusion. Taking Story County in Iowa, USA, as a case study, which covers the entire growth cycle of corn and soybeans, the study utilizes the random forest algorithm to evaluate the importance of eight vegetation indices (such as NDVI, NDRE) and five polarization features (VV, VH), selecting the optimal temporal combinations. Subsequently, thirty scenes of radar (Sentinel-1) and sixteen scenes of optical (Sen-tinel-2) images are fused at the feature level to construct a 46-dimension temporal feature map. Finally, a U-Net deep neural network is employed for refined crop classification, and the results are systematically compared with those from single-modality imagery.

The main innovations of this paper include the following: (1) the development of a multimodal remote sensing temporal feature selection and evaluation framework that integrates optical and radar data; (2) the proposal of a feature-level fusion strategy that systematically enhances the temporal representation and classification capabilities of multi-source remote sensing data; (3) the implementation of high-precision crop mapping through the integration of the U-Net deep learning model, with validation of its adaptability and robustness in a typical agricultural region. This study provides theoretical and methodological support for improving the accuracy of remote sensing-based crop classification and achieving precision agricultural management.

## 2. Advances in Crop Classification Using Remote Sensing

With the advancement of agricultural informatization and remote sensing technologies, fine-grained crop classification based on remote sensing images has emerged as a focal area of research. In recent years, the fusion of multimodal remote sensing data, such as optical and radar images, combined with temporal sequence features, has become a new means to enhance crop classification accuracy, further underscoring the pivotal role of remote sensing technology in agricultural remote sensing.

Current research trends are leaning towards process automation, region-adaptive models, and geographic knowledge-guided modeling. In terms of methodological evolution, crop classification has transitioned from traditional machine learning like SVM and RF to deep learning, such as CNN, LSTM, and U-Net, and its applications have also progressively expanded into fields like disaster monitoring, yield prediction, and resource assessment, further highlighting the comprehensive value of remote sensing technology in agriculture.

### 2.1. Progress in Crop Classification Using SAR Images

Synthetic Aperture Radar (SAR) images, which are not affected by weather conditions and provide round-the-clock imaging capabilities, have become an important data source for crop remote sensing identification; especially in areas with frequent cloud cover or high demands for timely data acquisition, capturing variations in backscatter caused by changes in canopy structure, moisture variation, and surface roughness during crop growth can provide rich temporal information for crop type identification [6]. SAR images provide various features for crop identification, including backscatter coefficients (σ^0^), polarization features (VV/VH), texture features, and coherence features, and among these, the VV polarization is indicative of vegetation canopy density, while the VH polarization is sensitive to vegetation structure, and texture features can improve crop classification accuracy to a certain extent [7].

In recent years, SAR temporal sequence features have been widely applied in crop growth cycle modeling and recognition identification, indicating that different crops exhibit certain patterns in their backscatter curves during their growth cycles [8]. Extracting changes in backscatter coefficients during the crop growth period can significantly enhance identification accuracy; for example, Sellaperumal et al. utilized multi-temporal RADARSAT images to extract backscatter curves for different crops, resulting in a notable improvement in classification accuracy [9]. The high revisit frequency of Sentinel-1 enables the identification of key growth stages, such as emergence and heading for various crops [10], and with the development of deep learning, convolutional neural networks (CNN) excel at extracting spatial and texture features, while LSTM networks demonstrate superior performance in modeling temporal changes in crop growth. Zhou et al. used LSTM to model the time series of Sentinel-1 VV/VH data, achieving the high-precision identification of corn and soybeans in North China [11]. Additionally, some studies have attempted to incorporate attention mechanisms to further enhance the model’s ability to extract critical temporal information. In China, many scholars have also proposed crop identification models more suitable for the regional context; for instance, Zhao et al. evaluated three deep learning models based on Sentinel-1A time series images, achieving satisfactory classification results in Zhanjiang, China [12]. Tian et al. achieved the high-precision identification of summer corn planting areas based on Sentinel-1A SAR images by extracting echo coefficients, synthesizing enhanced images, and using decision tree classification, with a validation accuracy of 96.55% [13]. Wei et al. employed a U-Net model with multi-temporal SAR images to realize large-scale crop mapping [14]. Andrimont et al. generated the first 10 m resolution continental-level crop type map for the EU, covering 19 crop types, using Sentinel-1 data from 2018 and the LUCAS 2018 survey with the RF algorithm. The results showed an overall accuracy from 76% to 80.3% for the map [15].

Despite the notable achievements made in SAR-based crop identification, challenges such as speckle noise, complex processing workflows, and poor model transferability still persist. Future efforts need to further improve the efficiency of SAR data preprocessing, develop more adaptable classification models, and promote their application in agricultural remote sensing.

### 2.2. Progress in Crop Classification Using Optical Images

Optical remote sensing images, characterized by their rich spectral information and high spatial resolution, exhibit excellent identification capabilities for different types of crops and can reflect physiological changes in crops at various growth stages [16]. Currently, major data sources for optical remote sensing include Landsat, MODIS, Sentinel-2, the GF series, and commercial satellites [17]. Among these, Sentinel-2, which features 13 spectral bands with different spatial resolutions (10 m, 20 m, and 60 m) and a five-day revisit cycle, is the most widely used in current research. Despite its relatively lower spatial resolution, MODIS, with its high temporal resolution (1–2 days), is widely used for large-scale applications. A small number of studies have leveraged high-resolution images acquired from unmanned aerial vehicles (UAVs) to achieve fine-scale classification within small areas.

Vegetation indices are commonly used spectral features in remote sensing crop monitoring. Among them, the Normalized Difference Vegetation Index (NDVI) effectively reflects crop growth conditions. The enhanced Vegetation Index (EVI), Red-Edge Normalized Difference Vegetation Index (NDRE), and Green Chlorophyll Index (GCI) are often used to characterize physiological parameters such as chlorophyll content, leaf area index, and nutritional status of crops. Notably, the red-edge bands carried by the Sentinel-2 satellite have demonstrated remarkable effectiveness in distinguishing crop types with similar spectral characteristics, becoming a key focus of research in recent years [18]. In the early stages of crop classification, pixel-level supervised classification methods, such as maximum likelihood classification (MLC), support vector machine (SVM), and decision tree (DT), were commonly employed. Among these, SVM was widely applied due to its stable performance with small samples, while random forest (RF) emerged as a common classification tool due to its strong ability to handle high-dimensional data. With the increasing availability of multi-temporal data, dynamic monitoring throughout the entire crop growth cycle has become a research trend, as the NDVI and EVI curve characteristics of different crops vary significantly at different growth stages. Modeling based on these temporal features enables the differentiation of food crops such as corn, wheat, and soybeans [19]. In recent years, deep learning methods have gradually been applied to crop identification using optical remote sensing images, with CNN, RNN, and LSTM network models improving identification accuracy through automatic feature extraction and temporal modeling. Currently, many research teams have conducted extensive studies based on remote sensing data from domestic GF satellites and Sentinel-2, focusing on improving regional classification accuracy, multimodal data fusion, and comparing classification algorithm performance. For example, Chen et al. utilized Sentinel-2 NDVI time series data and the RF model to identify winter wheat–summer maize double-cropping systems in the Huang-Huai-Hai Plain, achieving a classification accuracy of 93.4% [20]. Wang et al. constructed time series data of NDVI and humidity index, combined with RF, to achieve the fine mapping of land use in agricultural areas, with an overall accuracy of 88.87% [21]. Hu et al. proposed a crop remote sensing mapping method based on phenological and temporal feature selection, automatically selecting key features and removing redundancies by mining crop phenological information and generating high-quality corn planting maps for Heilongjiang Province using a support vector machine (SVM), with an accuracy exceeding 85% [22]. Zhang et al. analyzed the effectiveness of temporal and spectral features in crop classification using three methods, such as CART, SVM, and RF, with Sentinel-2 time series images from Yi’an County, Heilongjiang Province, with results showing that random forest achieved the highest classification accuracy (overall accuracy of 97.85% and Kappa coefficient of 0.95) [23].

Despite the widespread application of optical remote sensing in crop identification, challenges such as data loss during cloudy and rainy weather, the spectral overlap of the same crop at different growth stages, and the high cost of acquiring high-resolution data persist.

### 2.3. Progress in Crop Classification Using Multimodal Data Fusion

Given the issues of mismatched spatial resolutions and data gaps inherent in single remote sensing datasets, the fusion of multimodal remote sensing data has become an effective way to improve crop identification accuracy. Multimodal remote sensing fusion utilizes data from optical, SAR, thermal infrared, and hyperspectral sources to enhance classification stability and timeliness through their complementary spatial and informational characteristics, and optical data can reflect the internal physiological states of crops, such as chlorophyll content and vegetation indexes, while SAR data can penetrate clouds to obtain information on crop structure and moisture, offering significant advantages in cloudy and rainy regions. The fusion of these two types of data has become a mainstream research approach [24].

In the realm of crop identification research leveraging multimodal data fusion, numerous experiments have been applied in practical areas; for instance, Inglada et al. achieved the high-precision identification of major crops in agricultural areas of France by integrating Sentinel-1 and Landsat-8 data, incorporating object features and time series classifiers [25]. Liu et al., in 2021, constructed vegetation indices and backscatter curves based on Sentinel-1 and Sentinel-2 data, employing the random forest method to accomplish multi-crop classification in India [26]. Rao et al. further fused the temporal features of these two datasets, utilizing the random forest and LSTM models to classify crops in France, achieving an accuracy exceeding 90% [27]. Kussul et al. employed SAR, Landsat, and MODIS data in Ukraine to train a deep neural network for large-scale crop identification, demonstrating the significant advantages of multimodal data fusion in temporal modeling and model robustness [28]. Huang et al. utilized bidirectional LSTM to model NDVI and VV/VH, achieving the fine classification of crops such as corn and soybeans [29]. Zhi et al. extracted soybean planting areas by integrating Sentinel-1/2, MODIS and Landsat data on the GEE platform, combined with the random forest method, validating the reliability and scalability of this approach [30]. Zhou et al. successfully delineated the winter wheat planting range in the Huang-Huai-Hai Plain region using Sentinel-1/2 data and the random forest algorithms [31]. Wang et al. believed that optical and SAR data complement each other in terms of temporal sensitivity and adaptability to different weather conditions, and their fusion contributes to enhancing classification stability and accuracy [32]. Additionally, Xie et al. introduced the ST-CNN deep model, which fuses multi-temporal features from SAR and optical data, demonstrating superior classification performance in complex backgrounds [33]. Demarez et al. explored the early classification of irrigated corn crops in the temperate region of southwestern France by combining Sentinel-1, Landsat 8, and SRTM DEM data, showing that the RF classification method using multiple datasets (Kappa coefficient of 0.89) was more effective than using any single dataset alone (Kappa coefficient of 0.84) [34].

However, multimodal remote sensing data fusion still faces numerous challenges. For example, data preprocessing is complex, involving tasks such as registration and resampling, high-dimensional feature redundancy can lead to model overfitting, the model’s generalization ability is poor across different research areas, and the fusion of SAR speckle noise and optical texture features presents significant difficulties [35]. In the future, it is necessary to further strengthen the joint spatiotemporal modeling of multimodal data and optimize feature selection to enhance the model’s cross-regional stability and generalization ability. Meanwhile, integrating non-remote sensing data, such as meteorological, soil, and crop structure information, into fusion research should be pursued to promote crop classification toward large-scale, high-precision, and multi-dimensional development.

## 3. Materials and Methods

### 3.1. Overview of the Study Area

**Figure 1:**
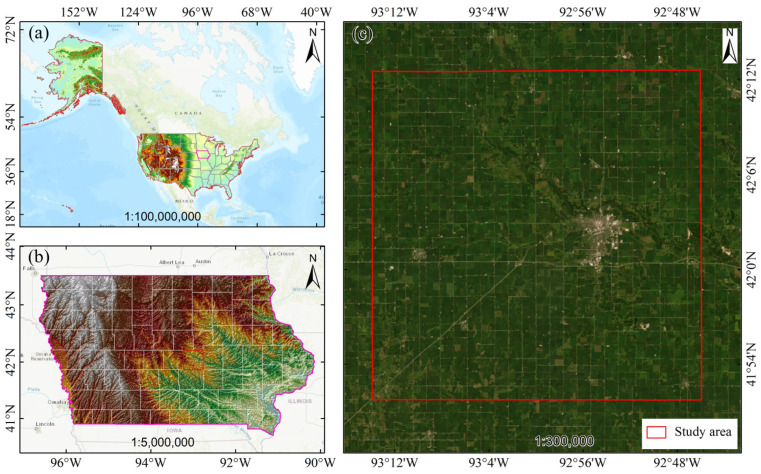
Overview Map of the study area: (**a**) Location of the study area within North America; (**b**) Topographical map of Iowa showing terrain features; (**c**) High-resolution satellite imagery of the study area.

Story County, located in central Iowa, is a typical agricultural county in the United States. The study area generally presents a rectangular shape, stretching approximately 48 km from north to south and about 30 km from east to west, with a total area of around 1490 square kilometers. The terrain is flat, with an average elevation of about 308 m, and the soil primarily consists of glacial deposits and loess, characterized by high organic matter content and good soil and water conservation capabilities. Survey data indicate that the Crop Suitability Rating (CSR) for cultivated crops in this area is generally high, with most scores scoring above 80, making it highly suitable for field crop cultivation. The county has a temperate continental climate with distinct seasons, and an average annual precipitation of about 914 mm, predominantly concentrated in the spring and summer when crops are in their active growth phases. The annual average temperature is about 10 °C, with July being the hottest and January the coldest. Despite the high level of agricultural development, the area still retains a certain amount of natural grasslands and wetlands, which help improve soil health and biodiversity [36]. The geographical location of the study area is illustrated in Figure 1. As a core region within the United States “Corn Belt”, agricultural land accounts for over 85% of the county’s total area, with corn–soybean rotation being the predominant farming practice, making it one of the main grain-producing areas in Iowa. Data obtained from 2022 indicate that the average corn yield in this area reached 13,230 kg/ha, and soybeans reached 4300 kg/ha, both higher than the state average [37].

### 3.2. Data Sources

#### 3.2.1. Sentinel-1 SAR Images

Sentinel-1, an SAR satellite under the European Space Agency’s Copernicus program for Earth observation, offers all-weather and day-and-night imaging capabilities, overcoming the limitations of optical remote sensing that are susceptible to cloudy and rainy conditions [38]. This study uses its Interferometric Wide (IW) mode, with an imaging width of 250 km and a spatial resolution of 10 m, featuring VV/VH dual-polarization, which can obtain information on crop structure and moisture status, making it suitable for large-scale crop monitoring. Sentinel-1 is commonly employed to construct backscatter coefficient curves for crops, to extract polarization and texture features, and to assist in identifying different crop types and growth stages. Its twin-satellite system provides a 6-day revisit cycle (3 days for cross-orbit configurations), facilitating dynamic monitoring and temporal analysis; with its high spatial and temporal resolutions, as well as polarization information, Sentinel-1 is widely applied in crop classification, area estimation, and soil moisture monitoring, offering crucial support for the feature extraction of corn and soybeans in this study.

#### 3.2.2. Sentinel-2 Optical Images

Sentinel-2 is a multi-spectral remote sensing satellite under the European Space Agency’s Copernicus program, consisting of two satellites, A and B, launched in 2015 and 2017, respectively, and its multispectral imager (MSI) is equipped with 13 spectral bands, covering the visible to shortwave infrared regions, with image resolutions of 10, 20, and 60 m [39]. Operating in tandem, the twin satellites provide a 5-day revisit cycle, making them suitable for various agricultural tasks. The Sentinel-2 data products mainly include two levels, L1C (raw images) and L2A (pre-processed images), with the latter being suitable for calculating vegetation indices such as NDVI, EVI, and NDRE, and they are widely used for crop growth monitoring and classification modeling [40]. The advantages of Sentinel-2 in agricultural remote sensing are mainly reflected in its high spatial resolution, dense temporal coverage, and rich spectral information, especially the red-edge and shortwave infrared bands, which make the classification of corn and soybeans and the differences in growth among different crops more pronounced. Table 1 presents the relevant parameters of the important bands for Sentinel-2.

#### 3.2.3. United States Corn Belt Data Layer (CDL)

**Figure 2:**
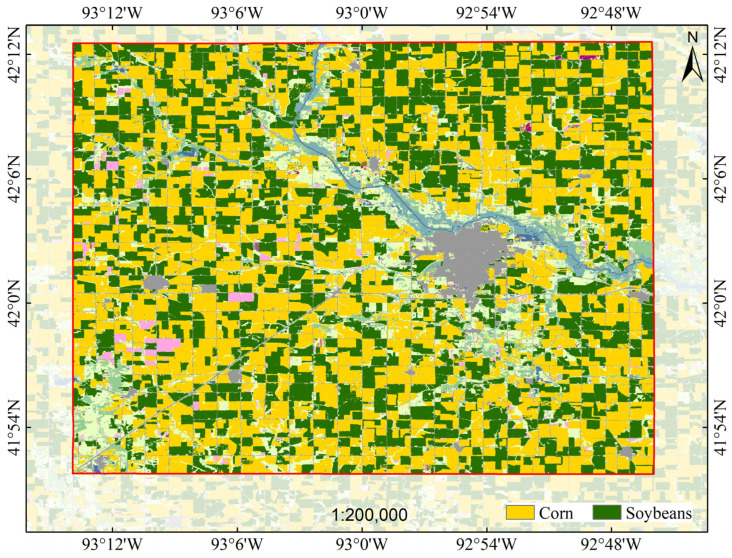
Distribution of corn and soybean crops in Story County in 2022.

The United States Corn Belt Data Layer (CDL) is a 30 m resolution crop distribution map generated through advanced algorithms based on multi-source remote sensing images (such as Landsat, MODIS, and Sentinel) and ground survey data. It is widely used for crop classification and agricultural monitoring. This dataset annotates the planting areas of major crops, including corn, soybeans, and wheat [41]. The data can be freely accessed at: https://nassgeodata.gmu.edu/CropScape/ (accessed on 18 May 2025). The distribution of different crops in Story County in 2022 is illustrated in Figure 2.

### 3.3. Data Preprocessing

#### 3.3.1. Sentinel-1 SAR Image Preprocessing

In this study, Sentinel-1 images provided by the European Space Agency were utilized, and preprocessing was conducted using the Google Earth Engine (GEE) platform. Compared to traditional software (such as SNAP and ENVI), GEE automatically completes orbit registration, radiometric calibration, geographic registration, and terrain correction, requiring only supplementary processing for speckle noise and certain details. The preprocessing workflow involved spatial cropping based on the vector boundary of the study area and nearest neighbor interpolation resampling to ensure consistency across multi-temporal data. Both VV and VH dual-polarization bands and incidence angle information were retained to extract multi-dimensional scattering features. To reduce radiometric differences between different orbits, a cosine correction model was applied to normalize the backscatter coefficients with respect to the incidence angle. For noise suppression, a Lee filter with a 7 × 7 window (with an equivalent number of looks, ENL, of 5) was employed to achieve a balance between noise suppression and edge feature preservation, and the linear intensity values were then converted to the dB scale using 10 × log^10^ transformation to optimize the input for classification algorithms. This process effectively improved the quality and usability of the SAR images, providing a solid data foundation for subsequent classification tasks.

#### 3.3.2. Sentinel-2 Optical Image Preprocessing

In this study, Sentinel-2 L2A optical images, which have undergone atmospheric correction, were employed and directly reflect surface reflectance and are suitable for vegetation monitoring and crop classification. Preprocessing was conducted using the GEE platform, and the Scene Classification Layer (SCL) was first utilized to mask out disturbances such as cloud shadows, medium-to-high probability clouds, and cirrus clouds, enhancing image clarity and increasing the proportion of usable pixels. Spatial clipping was then performed based on the study area boundary to ensure consistency in the range of multi-temporal images. To address the issue of inconsistent resolution among multispectral bands, the nearest neighbor interpolation method was used to resample the 20 m and 60 m bands to 10 m, achieving spatial alignment across multiple bands while preserving the original spectral characteristics, and this preprocessing workflow, which involves cloud detection, regional cropping, and resolution unification, improved image quality and provided highly consistent data support for subsequent classification and temporal analysis.

### 3.4. Random Forest (RF)

#### 3.4.1. Principle of Random Forest

Random forest (RF) is an ensemble learning method based on decision trees, proposed by Leo Breiman in 2001, and it is an implementation of the Bagging (Bootstrap Aggregating) paradigm, which constructs multiple decision tree classifiers. When making predictions, it combines the results of multiple estimators to produce the final output result [42], which enhances the overall model’s generalization ability and robustness, offering superior predictive performance compared to individual estimators while facilitating the parallelization of operations.

#### 3.4.2. Evaluation Metrics for Random Forest

To evaluate the classification effects of different backscatter feature combinations (like VV, VH, and VV/VH) and different vegetation index combinations (such as NDVI, EVI, and NDRE) from Sentinel-1 imagery, the confusion matrix [43] was used as the basic evaluation metric. Subsequently, metrics including overall accuracy (OA) and the Kappa coefficient were calculated to identify the optimal combination of backscatter features and vegetation indices, thereby improving the classification accuracy of major crops, such as corn and soybeans. The experiment was conducted on the Google Earth Engine (GEE) platform using the random forest feature importance evaluation, with the following two key parameters adjusted: (1) the number of trees (numberOfTrees) was set to 100 to ensure model stability, and (2) the minimum leaf population (minLeafPopulation) was set to 10 to reduce the risk of overfitting. All other parameters were kept at their default settings.

### 3.5. U-Net

#### 3.5.1. U-Net Principle

U-Net is a widely recognized convolutional neural network structure for image semantic segmentation, which first appeared at the MICROCAL Medical Image Analysis Conference in 2025 [44]. Initially developed for biomedical image analysis, U-Net has gained popularity in pixel-level classification tasks due to its simple structure, high training efficiency, and good generalization ability with limited sample data.

**Figure 3:**
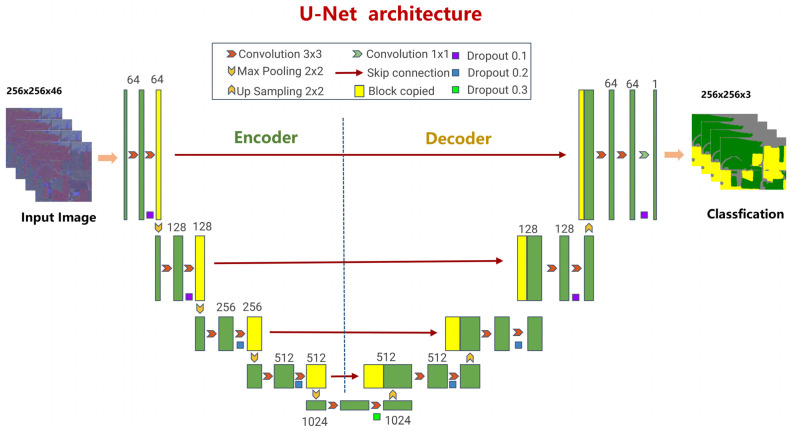
U-Net neural network structure.

The U-Net structure is symmetrically shaped like a “U” and is primarily composed of two main parts, the encoder (down-sampling part) and the decoder (up-sampling part). The encoder, mainly consisting of convolutional layers and max-pooling layers, gradually compresses the feature map scale while extracting high-level semantic features; the decoder, comprising up-sampling (transposed convolution) and convolutional layers, gradually restores the spatial resolution. A notable characteristic of this model is the incorporation of skip connections, which directly concatenate feature maps from the shallow layers of the encoder to the corresponding layers in the decoder, thus retaining more spatial detail information during the up-sampling process and improving segmentation performance [45]. Figure 3 illustrates the U-Net network structure.

#### 3.5.2. U-Net Classification Evaluation Metrics

By using the U-Net neural network for crop classification on fused multimodal remote sensing feature images, the following metrics are adopted for accuracy assessment to evaluate the performance of the final classification results:

(1) Precision: The proportion of actual samples belonging to the class among those predicted as the class, reflecting the “credibility” of the classification results. The formula is as follows:

(1) $$Precision_{i}=\frac{TP_{i}}{TP_{i}+FP_{i}}$$

(2) Recall: The proportion of samples that are actually of class A and are correctly identified by the model. The formula is as follows:

(2) $$Recall_{i}=\frac{TP_{i}}{TP_{i}+FN_{i}}$$

(3) F1-score: A harmonic mean that takes into account both precision and recall, making it suitable for classification scenarios where the distribution of class samples is imbalanced. The formula is as follows:

(3) $$F1_{i}=2\cdot \frac{Precision_{i}\cdot Recall_{i}}{Precision_{i}+Recall_{i}}$$

The parameters used in the above three formulas are as follows:

TP stands for true positives, predicted as positive and actually positive; FP stands for false positives, predicted as positive but actually negative; FN stands for false negatives, predicted as negative but actually positive; TN stands for true negatives, predicted as negative and actually negative.

## 4. Experimental Design

### 4.1. Principle of Crop Identification Using Multimodal Data

Fusing Sentinel-2 and Sentinel-1 images is a key method used to enhance the accuracy and stability of crop classification. Optical images are sensitive to vegetation chlorophyll and phenological changes, while radar images, unaffected by weather conditions, can capture crop structure and moisture content. The complementary nature of these two types of images facilitates a comprehensive portrayal of crop growth features, enhancing the discriminative ability of classification models.

**Figure 4:**
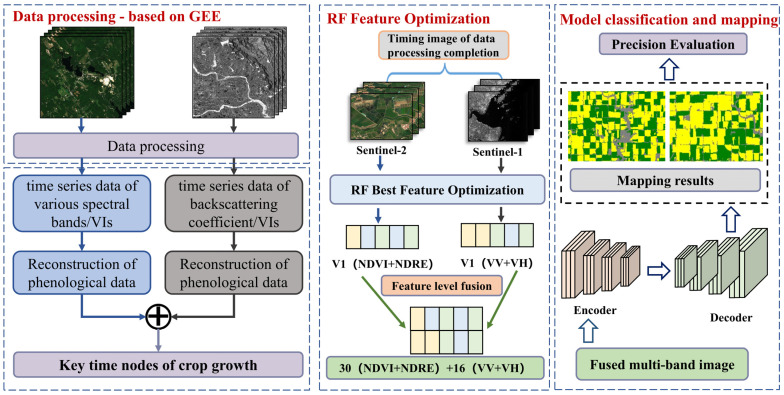
Feature extraction process based on optical and radar data.

The experimental findings reveal that corn and soybeans exhibit significant differences and separability in the temporal curves of vegetation indices such as NDVI and NDRE, as well as in the backscatter coefficient curves of C-band VV/VH polarization. Common strategies for multimodal remote sensing fusion include pixel-level, feature-level, and decision-level fusion, with feature-level fusion being the most suitable for this study’s needs, as it maximizes information retention and explores complementarity by concatenating feature vectors from different sources. Therefore, this experiment adopts a feature-level fusion strategy, constructing an optimal temporal feature input for both optical and radar data into a deep learning model to improve classification accuracy and robustness. The process of feature extraction and fusion is illustrated in Figure 4.

### 4.2. Optimization of Sentinel-1 Backscatter Features

This study focuses on corn and soybeans, analyzing their temporal backscatter coefficient characteristics throughout the growth cycle and extracting typical response curves under VV and VH polarizations. To evaluate the impact of different polarization combinations on classification accuracy, five feature combination schemes, including both VV and VH polarizations, were constructed on the GEE platform. Random forest was used as the classifier, and a comparative analysis of classification accuracy was conducted using confusion matrices to determine the best polarization feature combination. The specific feature combination schemes and classification accuracies are presented in Table 2.

The classification results indicate that, among single-polarization features, VH polarization outperforms VV polarization, as VH polarization is more sensitive to volume scattering from the crop canopy, which facilitates the differentiation between corn and soybeans, which exhibit distinct structural differences. The V1 (VV + VH) combination, which integrates surface scattering information from VV and volume scattering information from VH, achieves complementarity and enhances classification accuracy to 72.89%, making it the most effective combination. In contrast, combinations based on differences or ratios, such as V2 (VV − VH) and V3 (VV/VH), perform less favorably, especially V3, which has an accuracy of only 65.78%. This may be due to the operations disrupting the original polarization information, introducing noise, and compromising feature representation. In summary, the V1 combination preserves the original polarization information and achieves effective fusion, making it the best polarization combination for radar image experiments.

### 4.3. Sentinel-2 Vegetation Index Feature Selection

This study utilized Sentinel-2 L2A imaging to acquire optical data covering the study area from March to December 2022, constructing a time series feature set for crop classification. Three vegetation indices, namely NDVI, EVI, and NDRE, were selected to reflect the spectral changes of corn and soybeans. Eight feature combination methods, including these three vegetation indices, were constructed through mathematical operations. The GEE platform’s random forest classifier was employed for classification, with specific combinations and corresponding accuracies detailed in Table 3.

The classification results demonstrate that combining multiple vegetation indices enhances crop classification performance. Among them, V1 (NDVI + NDRE) achieves the highest overall accuracy by fully utilizing the complementary information from both indices, thereby enhancing the model’s ability to distinguish between crops. In contrast, the classification outcomes of V2 (NDVI + EVI) and V3 (NDRE + EVI) are slightly inferior, indicating that EVI has limited enhancement capability in the combination. Although V5 (NDVI × NDRE) has slightly lower accuracy than V1, it demonstrates the potential of nonlinear fusion in feature representation. In summary, V1 is the best feature combination for crop classification based on vegetation indices.

### 4.4. Feature Construction and Fusion

Through the previous feature selection, the following two types of multi-feature temporal sequence images were finally obtained: S1 (comprising 30 SAR images with VV + VH) and S2 (containing 16 optical images with NDVI + NDRE). Subsequent feature-level fusion was performed on these two multi-feature images, and the following are the relevant preprocessing operations:

(1) Temporal Alignment: Retain the respective temporal resolutions of the two data types, constructing a unified temporal sequence covering the entire growth cycle while preserving the original temporal resolution.
(2) Normalization: Perform [0, 1] normalization on all bands of the two images to eliminate numerical differences between data sources, ensuring that feature weights can achieve balance during model training. The normalization formula is as follows:

(4) $$X_{narm}=\frac{X-X_{min}}{X_{max}-X_{min}+\dot{0}}$$

where $\dot{0}$ is a small constant to prevent the denominator from being zero.

(3) Geometric Consistency Check: Ensure that the images are completely consistent in coordinate system, resolution, range, and transformation parameters.
(4) Feature Splicing: Splice optical features (NDVI + NDRE of 16-time nodes) and radar features (VV + VH of 30-time points) together to construct a 46-dimensional fused feature vector for subsequent classification modeling.

### 4.5. Classification Model and Training Strategy

This study employs the U-Net deep neural network for the classification of corn, soybeans, and others, and it conducts a series of optimizations on the model structure and training strategy for multimodal temporal features to enhance classification accuracy and training stability.

(1) Input Layer Adaptation: The U-Net input structure is modified to accommodate the 46-dimensional multimodal temporal feature vector.
(2) Loss Function Optimization: To address the issue of class imbalance, focal loss is adopted as a replacement for the standard cross entropy loss. Focal loss dynamically adjusts sample weights, effectively alleviating training bias caused by class imbalance and improving the model’s recognition capabilities for minority classes and difficult-to-classify samples.
(3) Optimizer and Learning Strategy: The Adam optimizer is employed, with an initial learning rate set at 1 × 10^4^. Combined with the ReduceLROnPlateau learning rate decay mechanism, the learning rate is dynamically adjusted based on the validation set loss to promote stable convergence.
(4) Early Stopping Strategy: A patience value of 15 is set. If the validation set loss does not show significant improvement for 15 consecutive rounds, training is terminated early to prevent overfitting and enhance efficiency.
(5) Model Saving and Evaluation: Both the “best model” based on validation set performance and the “final model” are saved, and a comprehensive evaluation is conducted using multiple metrics, including accuracy, precision, recall, and F1 score.

## 5. Experimental Results and Analysis

This experiment was conducted in Story County, Iowa, USA, following a three-step process of feature selection, fusion, and classification in sequence. Firstly, the optimal vegetation indices and polarization features were screened using RF; secondly, feature-level fusion was used to construct multi-feature remote sensing images; finally, crop classification was performed within the U-Net framework to distinguish corn (0), soybeans (1), and others (2). The image data underwent normalization, label re-encoding, and spatial registration before being cropped into 256 × 256 pixel tiles, divided into training, validation, and test sets in a 6:2:2 ratio. The experimental parameters were set as follows: 100 iterations, a batch size of 16, an initial learning rate of 1 × 10^4^, using Early Stopping (patience = 15), and the best model saving mechanism. To validate the fusion effect, classification results obtained from fused data were compared with those from single data sources (S1 and S2). In addition, to further evaluate the performance and reliability of the proposed U-Net model, seven representative models were selected for comparison, including traditional methods (SVM, RF), classical deep learning models (FCN, SegNet), and temporal modeling approaches (TCN, ConvLSTM, SegFormer).

### 5.1. Classification Results and Accuracy Assessment of the Optimal Sentinel-1 SAR Temporal Features

**Figure 5:**
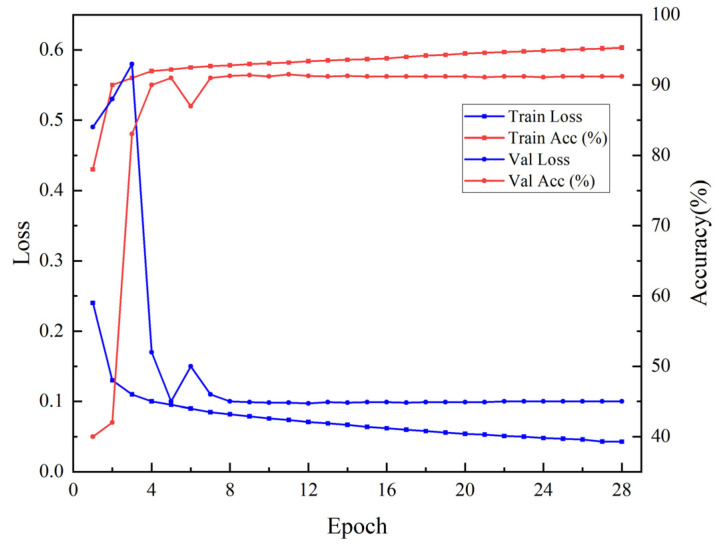
Loss during Sentinel-1 training process and accuracy during Sentinel-1 training process.

In this section, the U-Net model was trained for crop classification based on the optimal temporal features V1 (VV+VH) derived from S1 SAR images. As illustrated in Figure 5, the training loss continuously decreased with increasing epochs, indicating that the model was gradually fitting the data. The validation loss fluctuates significantly in the early stages, peaking at around 0.58 in the third epoch, before rapidly declining and stabilizing around 0.10 after the tenth epoch, suggesting that the model’s generalization ability was gradually improving. The training was automatically terminated at the 28th epoch due to the Early Stopping mechanism, effectively avoiding overfitting. Figure 5 displays the following changes in training and validation accuracy: the training accuracy increased from 78% to 95%, while the validation accuracy initially fluctuated before stabilizing at around 91% after the tenth epoch. Overall, the model performed consistently on both the training and validation sets, with a stable training process and reliable classification results, indicating good generalization ability.

**Figure 6:**
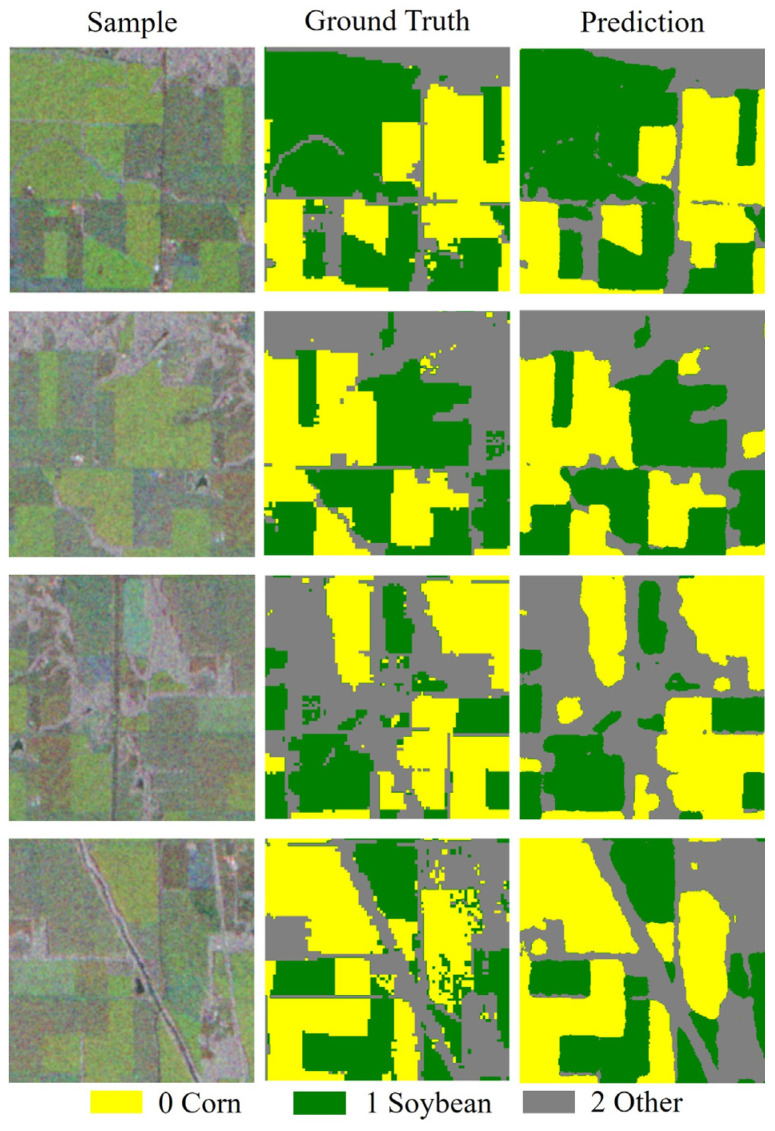
Sentinel-1 ground truth samples and predicted values.

Visualization results from Figure 6 indicate that, in large continuously cultivated areas, the model’s predictions align closely with the actual labels, especially in the first and second-row samples, where corn (yellow) and soybean (green) areas are accurately identified, indicating the effectiveness of SAR data in crop recognition. However, certain classification errors are observed at the edges of farmland, manifesting as blurred boundaries or misclassifications. The recognition performance for the “other” category (gray) is relatively weaker, with some non-agricultural areas (such as roads and construction sites) being misclassified as crops, which is particularly evident in the fourth-row sample, reflecting certain limitations of SAR data in distinguishing non-agricultural features. Overall, the model can accurately distinguish major crop areas but still exhibits confusion at blurred boundaries and where categories overlap, especially showing deficiencies in the “other” category.

Table 4 delineates the accuracy and loss changes of the model during the training, testing, and validation processes. Specifically, the training set exhibited an average loss of 0.0428, corresponding to an accuracy rate of 95.31%; the validation set loss was 0.0998, with an accuracy of 91.20%; and the test set showed a loss of 0.1178 and an accuracy of 89.25%. This result indicates the model’s stable convergence during training and its robust generalization ability, with no signs of overfitting. Notably, the accuracy on the test set approaches 90%, achieving good results under the condition of using only SAR data.

Table 5 presents the accuracy evaluation metrics for various crop types. The results indicate that the model performs relatively consistently for the corn and soybean categories, with both precision and recall exceeding 0.9, and F1-scores of 0.9060 and 0.9017, respectively. In contrast, the F1-score for the “Other” category is slightly lower (0.8642), primarily due to the internal diversity of land cover types within this category and the subtle differences in structural features of crops in the SAR image.

In summary, utilizing the optimal temporal features of Sentinel-1 SAR data within the U-Net model can effectively distinguish among corn, soybean, and other three categories, with excellent identification accuracy for corn and soybean, demonstrating the potential of radar data in discerning crop structures and spatial patterns. However, SAR features exhibit certain limitations when dealing with the “others” category, which has high spectral similarity or significant structural changes. Therefore, it is imperative to further introduce optical remote sensing features for multimodal fusion in subsequent research to enhance the model’s discriminative capability for fine categories and improve overall classification accuracy.

### 5.2. Classification Results and Accuracy Assessment of the Optimal Sentinel-2 Optical Temporal Features

**Figure 7:**
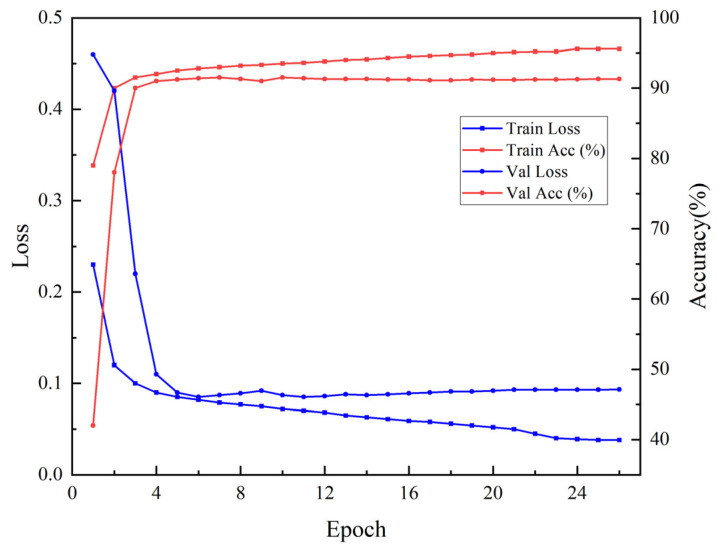
Loss during Sentinel-2 training process and accuracy during Sentinel-2 training process.

In this section, the classification training was conducted using the U-Net model with the optimal vegetation index temporal features extracted from S2 optical images, with model parameters set consistent with the previous section. As shown in Figure 7, the training loss decreased rapidly and stabilized at a lower level after the tenth round, indicating that the model fitted well on the training set. The validation loss exhibited significant fluctuations in the early stages, possibly due to unstable parameters or differences in the distribution of the validation set, but it stabilized at approximately 0.1 from the seventh epoch onward, with no overfitting observed. The training was reasonably terminated when Early Stopping was triggered at the 26th epoch. Figure 7 illustrates that the training accuracy stabilized at around 95% after the tenth round, while the validation accuracy remained around 92% from the fifth epoch onwards, with minor fluctuations. The stable improvement in validation accuracy reflects the model’s ability to effectively capture key features and prevent overfitting, which can be further optimized through parameter tuning or the introduction of regularization techniques.

**Figure 8:**
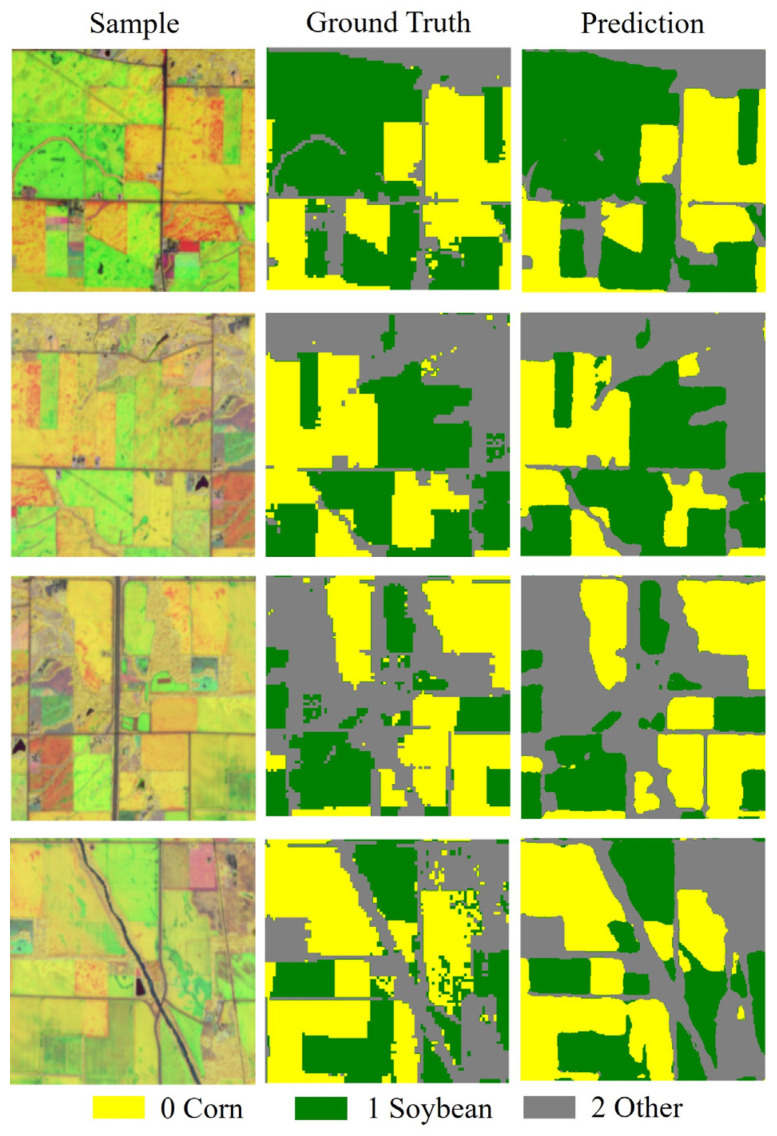
Ground truth samples and predicted values of Sentinel-2.

Figure 8 shows the classification results of typical plots using the optimal S2 optical temporal feature images. From left to right, the images depict the original image, ground truth labels, and the model’s predicted map. The results indicate that the model demonstrates high accuracy in identifying the two main crops, corn and soybeans, especially in large and regularly distributed farmland areas, where the predicted results align closely with the ground true labels, exhibiting intact block structures and clear boundaries. Despite some misclassifications in complex areas at crop boundaries and regions with non-crop interferences such as buildings and roads, which mainly manifest as blurred edges and misclassification of small patches, the overall prediction effect of S2 is favorable compared to the S1 classification results, strongly validating the significant improvement in model performance achieved by introducing optical temporal features.

As observed from Table 6, the model achieved a high accuracy of 95.59% on the training set, with accuracy rates of 91.3% and 90.36% on the validation and test sets, respectively, both of which are better than the performance when only using SAR features in the previous section. This indicates that optical temporal features possess strong discriminative power in crop identification, especially leveraging the advantages of vegetation indices like NDVI and NDRE, which reflect long-term crop growth conditions, thereby helping the model in accurately distinguishing crop categories.

In Table 7, both the precision and F1-score for the corn category exceed 0.91, with a recall of 0.9087, indicating the model’s most stable performance in corn identification. The various metrics for the soybean category are also quite balanced, with an F1-score of 0.9073, demonstrating good classification consistency. In contrast, although the F1-score for the “other” category is slightly lower (0.8835), it still remains at a relatively high level. Overall, the model achieves high accuracy across all three crop categories, with optical temporal features showing stronger discrimination ability in crop classification, especially in distinguishing between easily confused crops like corn and soybean, where the classification performance is significantly better than that of the SAR feature model.

Overall, the model based on Sentinel-2 optical features outperforms the model using only SAR data in terms of classification accuracy, especially showing significant improvements in accuracy for the dominant crop categories (corn and soybeans), reflecting the important value of optical remote sensing in crop classification. Future research could further explore the integration of optical and SAR data to balance spatial structure and spectral temporal information, thereby further enhancing classification accuracy.

### 5.3. Crop Classification Results Based on Fused Multimodal Remote Sensing Temporal Features

Building on previous experiments, this section introduces the 46-dimensional multimodal remote sensing temporal features that fused both S1 and S2 data, training a U-Net model for crop classification. The parameter settings remain consistent, and the model converges at the 27th epoch, yielding classification results for three land cover categories, followed by accuracy evaluation and visualization analysis.

**Figure 9:**
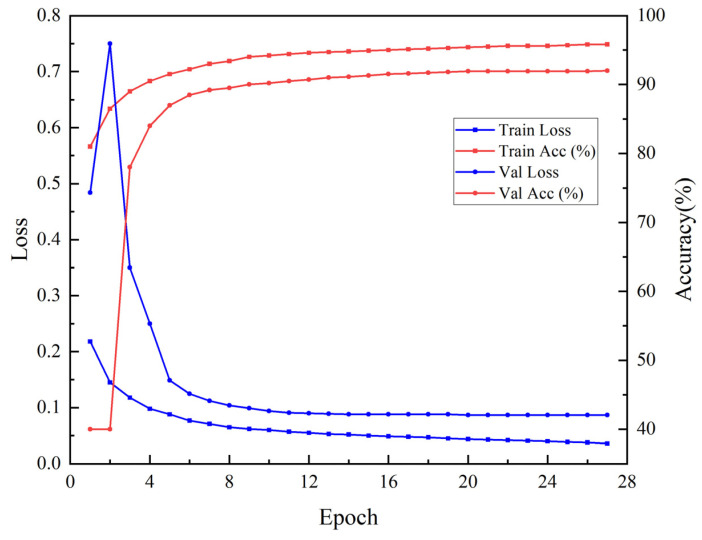
Loss during the training process of the fused images and accuracy during the training process of the fused images.

Figure 9 shows that the training loss continuously decreases and eventually stabilizes at around 0.04. The validation loss fluctuates significantly in the early stages, rapidly decreasing and stabilizing at around 0.09 by the fifth epoch, indicating good convergence of the model with no significant overfitting. The training was reasonably terminated at the 27th epoch due to the Early Stopping mechanism. In Figure 9, the training accuracy rapidly increases from about 81% to 92% in the first five epochs and finally approaches 95%. The validation accuracy rises sharply from 40% to 87% and stabilizes at around 92% after the tenth epoch. The small gap between training and validation accuracy indicates that the fused features effectively enhance the model’s discriminative ability and robustness.

**Figure 10:**
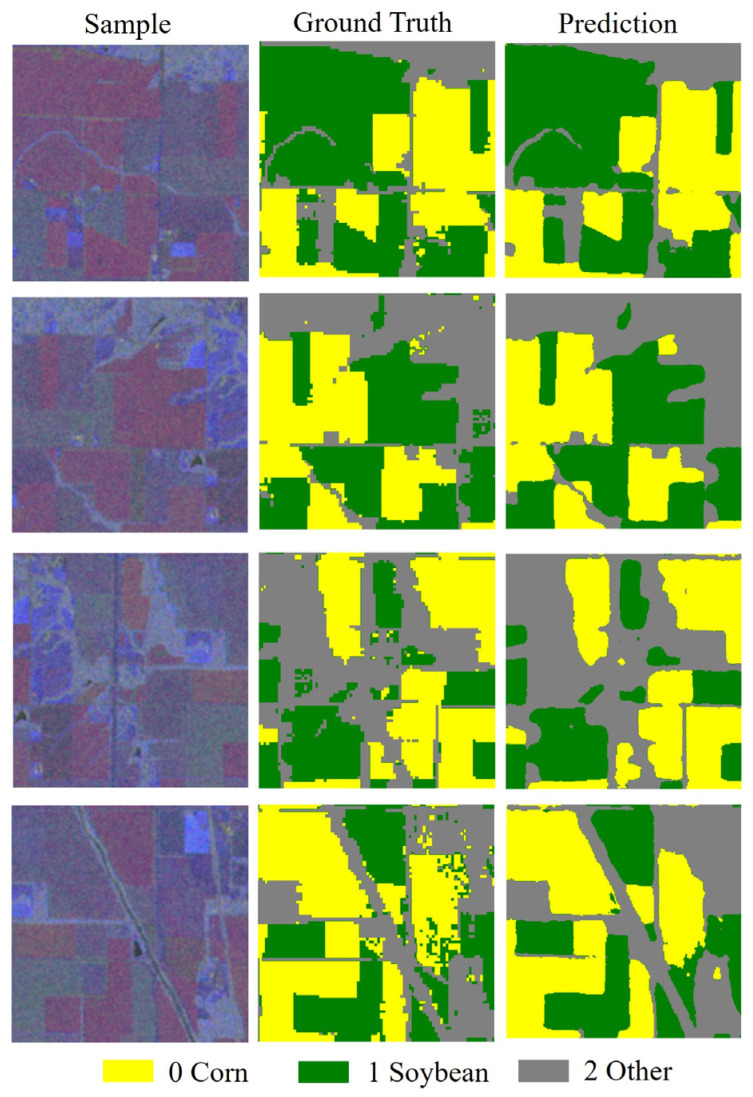
Ground truth samples and predicted values of the fused images.

A comparative analysis of the classification results in Figure 10 reveals that the fusion of multimodal remote sensing temporal features significantly enhances the overall performance of crop classification. When relying solely on SAR data, the classification results suffer from issues such as salt-and-pepper noise, blurred field boundaries, and poor spatial continuity; while optical temporal data offer good spectral separability, misclassifications still occur in areas with similar spectral characteristics of land covers or complex boundaries. After fusing the temporal features of optical and SAR data, the model demonstrates improved performance in identifying main crops. For example, the small corn field, which was originally difficult to identify, can be accurately extracted after fusion. The soybean cultivation areas in the third and fourth rows have more complete contours, with reduced fragmentation, and the overall classification results more closely resemble the actual ground distribution, featuring clearer boundary delineation.

Overall, the fused model aligns closely with the ground truth labels. Although there are some local misclassifications in a few complex backgrounds, it demonstrates that the fusion of multimodal remote sensing temporal features has a significant effect and great potential in improving crop classification accuracy.

As observed from Table 8, the model automatically stopped training at the 27th epoch, at which point the average loss value on the training set was 0.0362, with an accuracy of 95.83%; the loss for the validation set was 0.0868, with an accuracy of 91.99%; and the accuracy for the test set was 90.81%, with a loss value of 0.0961. The overall trend indicates that the model effectively converged during the training phase and possesses good generalization ability. Although the training set’s loss is significantly lower than those of the validation and test sets, the difference falls within a reasonable range, suggesting no significant overfitting.

Table 9 presents the classification evaluation metrics for the three land cover categories. The model demonstrates the best performance in corn identification, with an F1-score of 0.9211, indicating good stability for the main crops. For soybeans, although the category achieves the highest accuracy, its recall is slightly lower, resulting in an F1-score of 0.9133, which still reflects a solid overall good performance. Regarding the “other” category, despite a slightly lower precision (0.8790), both precision and recall remain above 0.88, indicating that the model exhibits a certain reliability in identifying land cover types within complex backgrounds. Overall, the model achieves high classification accuracy across three types of crops, especially for the two main crops, corn and soybeans, which perform better than earlier models that only used SAR or optical data. The introduction of multimodal remote sensing temporal features not only improves the overall classification accuracy but also enhances spatial consistency and boundary clarity.

### 5.4. Comparative Evaluation of Models on Multimodal Time Series Features

To validate the classification performance of the proposed U-Net model on multimodal remote sensing time series imagery, a comparative analysis was conducted involving seven widely used models: support vector machine (SVM), random forest (RF), fully convolutional network (FCN), SegNet, temporal convolutional network (TCN), ConvLSTM, and SegFormer. All models were trained using the same fused 46-dimensional feature maps as input, with the dataset split into training, validation, and testing subsets at a ratio of 6:2:2. The classification performance of each model was assessed across the three categories of corn, soybean, and others. Evaluation metrics included precision, recall, F1-score, and overall accuracy (OA).

Table 10 presents a comparison of the classification performance of various models on the testing dataset. Overall, the U-Net model demonstrates superior F1-scores across the three land cover categories, achieving 0.92 for corn, 0.91 for soybean, and 0.89 for the other category, with an overall accuracy (OA) of 91%. This performance slightly surpasses that of SegFormer, ConvLSTM, and TCN, reflecting U-Net’s strong capability in spatial structure preservation and semantic segmentation under multimodal fusion. Traditional models such as SVM and RF perform relatively poorly, with F1-scores below 0.88 and OA values of 86% and 84%, respectively. FCN, with its relatively shallow network architecture, attains an OA of 87%, demonstrating effective end-to-end modeling ability. SegNet and TCN, which integrate deep structures with temporal modeling, achieve an OA of 90%, exhibiting better boundary delineation and spatial consistency. SegFormer, leveraging Transformer-based global context modeling, maintains stable performance in the corn and soybean categories, with an OA of 91%. ConvLSTM, through its temporal modeling capability, delivers performance comparable to U-Net. In summary, U-Net achieves the best balance between accuracy and spatial consistency, producing more coherent classification boundaries and effectively adapting to the complex spatiotemporal characteristics of multimodal remote sensing data.

**Figure 11:**
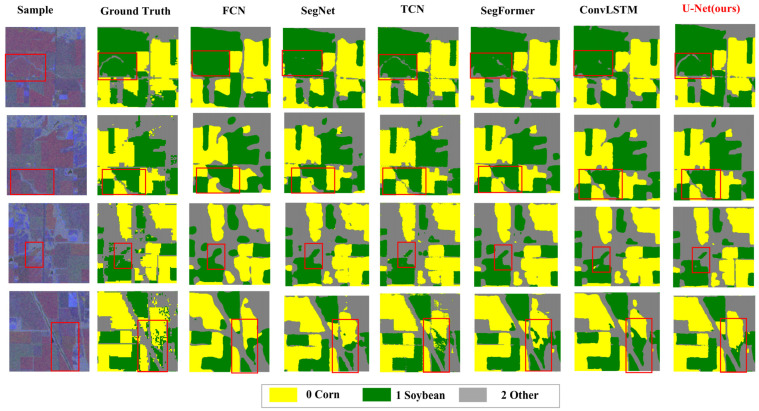
Visual comparison of different deep learning models.

Figure 11 presents the visualized classification results of the various models. The U-Net output demonstrates clear boundaries, high spatial consistency, and minimal noise. In contrast, the classification results of SVM and RF appear fragmented. FCN and SegNet improve detail preservation, but their boundaries remain blurred. Benefiting from temporal modeling, TCN and ConvLSTM perform well in spatial continuity. SegFormer achieves excellent overall performance, although deficiencies persist in areas with complex boundaries. Overall, U-Net achieves the best balance among classification accuracy, spatial consistency, and boundary clarity, confirming its advantage in processing multimodal remote sensing time series data.

### 5.5. Summary of This Section

A comparison was conducted among three schemes using single-modal features (S1, S2) and fused multimodal features. The results show that the model employing fused multimodal features performs more stably during the training, validation, and testing phases, achieving a testing accuracy of 90.81%. The single SAR model, while advantageous in plot structure recognition, suffers from blurred boundaries and excessive salt-and-pepper noise. The optical model excels in spectral discrimination but is prone to misclassification in areas with complex boundaries or similar spectra. The fused model effectively combines the SAR’s sensitivity to structural information with the optical data’s spectral advantages, significantly improving classification accuracy and spatial consistency. Finally, to validate the effectiveness of the U-Net model used in this experiment, it was compared with seven classical networks. The results show that U-Net achieves the best classification accuracy and spatial consistency under multimodal fusion, fully demonstrating its efficient capture of complex spatiotemporal features and outstanding generalization performance.

## 6. Conclusions and Future Work

This study focused on Story County, Iowa, USA, and aimed to address the limitations in crop classification accuracy caused by using a single remote sensing data source. Utilizing time-series Sentinel-1 SAR and Sentinel-2 optical images, we developed a crop classification approach that integrates multimodal remote sensing features with deep learning techniques. Specifically, the methodology includes the following components: (1) We proposed a framework to select and evaluate multimodal time-series features. Based on this, we identified the best SAR polarization combination (VV+VH) and the best optical vegetation index combination (NDVI+NDRE). (2) To combine information from different sources, we designed a feature-level fusion strategy. Selected features were stacked along the channel dimension, then standardized and spatially aligned to form a 46-dimensional time-series image. This process effectively merged structural features from SAR and spectral features from optical data. (3) Based on the U-Net deep learning model, high-accuracy crop classification was performed and compared with seven classical models, achieving an overall accuracy of 91%, with superior performance in spatial consistency and boundary delineation. The results demonstrate that the proposed method exhibits strong adaptability and application potential in multi-source data fusion and large-scale crop mapping.

Although the proposed method achieved satisfactory classification performance, several aspects remain to be further optimized. Firstly, while the U-Net model excels in spatial segmentation, it lacks the ability to effectively model temporal dynamics, limiting its capacity to fully capture the growth processes of crops. Future research may incorporate network architectures with temporal modeling capabilities, such as TCN, ConvLSTM, or Transformer, to enhance the temporal expressiveness of the model. Secondly, the current feature fusion approach is relatively simplistic, relying solely on channel-wise stacking to integrate multi-source information. This method does not incorporate attention mechanisms or dimensionality reduction strategies, which may lead to information redundancy and overfitting risks. Subsequent work could explore feature weighting mechanisms (e.g., SE modules, CBAM), principal component analysis (PCA), or automated feature learning modules to improve fusion efficiency and model robustness. In addition, biophysical variables such as LAI and biomass could be introduced as auxiliary features to further enhance crop separability. Future studies should also validate the adaptability of the proposed method across different geographic regions and cropping structures. By incorporating transfer learning or domain adaptation strategies, the model’s generalization capability and practical utility can be further strengthened, providing more reliable and efficient technical support for precision agriculture using remote sensing.

## Figures and Tables

**Table 1:** Sentinel-2 band information.

Band Name	Center Wavelength (nm)	Spatial Resolution (m)	Main Uses
Blue Light (B2)	490	10	Atmospheric correction, preliminary analysis of vegetation identification
Green Light (B3)	560	10	Vegetation status monitoring
Red Light (B4)	665	10	Calculation of NDVI and other vegetation indices in conjunction with near-infrared
Near Infrared (B8)	842	10	Monitoring of crop growth status, NDVI calculation
Red Edge (B5–B8A)	705–865	20	Chlorophyll content reflection, NDRE index construction
SWIR (B11/B12)	1610/2190	20	Soil moisture, vegetation stress identification

**Table 2:** Different polarization combination schemes and classification accuracies.

Feature Combination Method	Overall Accuracy (OA)	Kappa Coefficient
VV	0.8205	0.7290
VH	0.8886	0.8317
**V1 (VV + VH)**	**0.9059**	**0.8579**
V2 (VV -VH)	0.7598	0.6378
V3 (VV/VH)	0.6512	0.4764

**Table 3:** Vegetation index combination methods and classification accuracies.

Feature Combination Method	Overall Accuracy (OA)	Kappa Coefficient
NDVI	0.9262	0.8888
NDRE	0.9247	0.8867
EVI	0.9117	0.8671
**V1 (NDVI + NDRE)**	**0.9291**	**0.8932**
V2 (NDVI + EVI)	0.9016	0.8517
V3 (NDRE + EVI)	0.9059	0.8584
V4 (NDVI + NDRE + EVI)	0.9117	0.8670
V5 (NDVINDRE)	0.9233	0.8845

**Table 4:** Accuracy and loss metrics during Sentinel-1 training process.

28 Epochs Automatic Stop	Loss (Average)	Accuracy
Training	0.0428	95.31%
Test	0.1178	89.25%
Val	0.0998	91.2%

**Table 5:** Sentinel-1 accuracy evaluation metrics.

Category	Precision	Recall	F1-Score
0 (Corn)	0.91	0.91	0.91
1 (Soybean)	0.91	0.90	0.90
2 (Others)	0.86	0.87	0.86

**Table 6:** Accuracy and loss during Sentinel-2 training process.

26 Epochs Automatic Stop	Loss (Average)	Accuracy
Training	0.038	95.59%
Test	0.099	90.36%
Val	0.0932	91.3%

**Table 7:** Sentinel-2 accuracy assessment metrics.

Category	Precision	Recall	F1-Score
0 (Corn)	0.93	0.91	0.92
1 (Soybean)	0.91	0.91	0.91
2 (Others)	0.88	0.89	0.88

**Table 8:** Training process loss of fused images.

27 Epochs Automatic Stop	Loss (Average)	Accuracy
Training	0.0362	95.83%
Test	0.0961	90.81%
Val	0.0868	91.99%

**Table 9:** Accuracy assessment metrics for fused images.

Category	Precision	Recall	F1-Score
0 (Corn)	0.91	0.93	0.92
1 (Soybean)	0.93	0.90	0.91
2 (Others)	0.88	0.89	0.88

**Table 10:** Comparative classification performance of different models using multimodal remote sensing time series features.

Model	Corn			Soybean			Others			
	P	R	F1	P	R	F1	P	R	F1	OA
**SVM**	0.87	0.90	0.88	0.88	0.85	0.87	0.79	0.78	0.79	0.86
**RF**	0.86	0.87	0.86	0.86	0.84	0.85	0.80	0.80	0.80	0.84
**FCN**	0.90	0.88	0.89	0.88	0.88	0.88	0.83	0.85	0.84	0.87
**SegNet**	0.90	0.92	0.91	0.91	0.89	0.90	0.87	0.87	0.87	0.90
**TCN**	0.91	0.93	0.92	0.92	0.89	0.91	0.87	0.88	0.87	0.90
**SegFormer**	0.93	0.91	0.92	0.91	0.91	0.91	0.87	0.89	0.88	0.91
**ConvLSTM**	0.92	0.91	0.92	0.92	0.90	0.91	0.86	0.89	0.87	0.90
**U-Net (ours)**	0.91	0.93	0.92	0.93	0.90	0.91	0.88	0.89	0.89	0.91

**Note:** P denotes precision, R denotes recall, F1 denotes F1-Score, and OA stands for overall accuracy.

## Data Availability

The data presented in this study are available from the corresponding author upon request.
